# Types of Privacy Expectations

**DOI:** 10.3389/fdata.2020.00007

**Published:** 2020-02-25

**Authors:** Ashwini Rao, Juergen Pfeffer

**Affiliations:** Technical University of Munich, Munich, Germany

**Keywords:** data privacy, expectation, regulation, usability, empirical, user study

## Abstract

Understanding user privacy expectations is important and challenging. General Data Protection Regulation (GDPR) for instance requires companies to assess user privacy expectations. Existing privacy literature has largely considered privacy expectation as a single-level construct. We show that it is a multi-level construct and people have distinct types of privacy expectations. Furthermore, the types represent distinct levels of user privacy, and, hence, there can be an ordering among the types. Inspired by expectations-related theory in non-privacy literature, we propose a conceptual model of privacy expectation with four distinct types – Desired, Predicted, Deserved and Minimum. We validate our proposed model using an empirical within-subjects study that examines the effect of privacy expectation types on participant ratings of privacy expectation in a scenario involving collection of health-related browsing activity by a bank. Results from a stratified random sample (*N* = 1,249), representative of United States online population (±2.8%), confirm that people have distinct types of privacy expectations. About one third of the population rates the Predicted and Minimum expectation types differently, and differences are more pronounced between younger (18–29 years) and older (60+ years) population. Therefore, studies measuring privacy expectations must explicitly account for different types of privacy expectations.

## 1. Introduction

Internet, mobile applications and Internet-of-Things technologies have enabled collection and use of unprecedented amount of user data. Companies collect, share and combine large amount of user data including sensitive data related to personal health, income and religion (Rao et al., 2014). Such data practices often violate users' privacy expectations regarding products and services (Lin et al., 2012; Martin and Shilton, 2016b; Rao et al., 2016). For instance, 90% of the participants in a study did not expect banks to collect users' health information although banks do so (Rao et al., 2016). Even participants who were customers of the banks that collected users' health information did not expect it (Rao et al., 2016). Expectations influence decision making (Hogarth, 1987), and mismatches in expectations can adversely impact privacy decision making.

In order to improve consumer data privacy, regulatory agencies have sought to understand user privacy expectations (Council of The European Union, 2016; The U.S. Federal Trade Commission, 2019). The European Union General Data Protection Regulation (GDPR), which came into effect in 2018, emphasizes that companies should consider and carefully assess user privacy expectations.

“taking into consideration the **reasonable expectations** of data subjects” (Council of The European Union, 2016)

“[…] careful assessment including whether a data subject can **reasonably expect** at the time and in the context of the collection of the personal data that processing for that purpose may take place” (Council of The European Union, 2016).

Hence, complying with and enforcing GDPR depends on the ability to understand user privacy expectations, which requires accurately eliciting and measuring privacy expectations.

Empirical studies that measure privacy expectations (Lin et al., 2012; Martin and Shilton, 2016a,b) have largely considered privacy expectation as a single-level construct. Theoretical work on the conceptual definition of privacy expectation (Altman, 1975; Nissenbaum, 2009; Lin et al., 2012; Martin, 2016) has also largely considered privacy expectation as a single-level construct. In general, the privacy domain does not treat privacy expectation as a multi-level construct with different types of privacy expectations (Rao et al., 2016).

In contrast to the privacy domain, Consumer Satisfaction/Dissatisfaction (CS/D) and service quality domains treat expectation as a multi-level construct (Miller, 1977; Gilly et al., 1983; Zeithaml et al., 1993). CS/D literature supports four types of **consumer expectations**: *Ideal, Expected, Deserved*, and *Minimum tolerable* (Miller, 1977; Gilly et al., 1983). Service quality literature supports three types of **service expectations**: *Desired, Adequate*, and *Predicted* (Zeithaml et al., 1993). In these domains, expectations are considered as standards against which product performance and service quality are judged. Different types of expectations are different standards for customer assessment of satisfaction and quality. Distinguishing between types of expectations is important for measuring customer satisfaction and service quality.

Inspired by the work in Consumer Satisfaction/Dissatisfaction and service quality domains, in this work, we propose a conceptual model for privacy expectation as a multi-level construct. We use empirical evidence to show that people have distinct types of privacy expectations. The empirical evidence refutes the existing notion that privacy expectation is a single-level construct. This result has an important implication for measuring privacy expectations: studies have to explicitly identify and elicit the type of privacy expectation that is relevant for the study. Simply asking what users expect without differentiating between expectation types can lead to ambiguity. We summarize our **main contributions** as follows:

- We propose a conceptual model that treats privacy expectation as a multi-level construct. The model proposes four **types** of privacy expectations: *Desired, Predicted, Deserved* and *Minimum*. The types represent distinct levels of user privacy, and, hence, there can be an **ordering** among the types. The conceptual model is a contribution to privacy theory.
- We design and implement an empirical study for measuring the four types of privacy expectations. The study tests the validity of the conceptual model. It can inform the design of future studies for measuring privacy expectations accurately. Results from the study support the conceptual model.

## 2. Scope of the Work

In this work we address theoretical and empirical questions related to privacy expectations of users. We focus on expectations related to *informational privacy* or *data privacy* and not on other conceptualizations of privacy such as privacy as a right to intimate decisions about one's body or sexuality. Informational privacy (Westin, 2003) is related to collection, use, sharing, retention etc. of users' data by products and services. Products and services generally describe their data practices in a privacy policy. If products and services collect data regarding a person's sexuality or intimate decisions, then such data practices fall under the scope of informational privacy.

We do not focus on legal doctrines such as *right to privacy* or *expectations of privacy* as defined in the law. Legal doctrines and laws related to privacy vary widely across the world. It is beyond the scope of our work to explain how they may be related to this work. The results from our work, however, could be used to understand whether privacy laws and doctrines are grounded in users' expectations related to informational privacy.

## 3. Background

Social research methods use a two-phase approach consisting of an induction phase followed by a deduction phase to formulate and validate a conceptual model (Martin, 2007). The induction phase goes from one or more specific observations to a more general theory regarding a conceptual model. The deduction phase generates hypotheses based on the general theory and uses empirical evidence to validate the conceptual model. In this section, we discuss the context and observations that helped us formulate a conceptual model for privacy expectation presented in section 5. We discuss the empirical study we used to validate the conceptual model in section 6.

### 3.1. Expectation-Related Theory in Privacy Literature

In a prior empirical study related to privacy expectations (Rao et al., 2016), we had asked the participants “Do you **expect** the website to ask for your consent for sharing your information?” In an in-person interview, one participant had replied as follows:

“I think the expect question is a little hard to answer because I am thinking whether you are asking me what I think should be done or what I perceive how they are doing it now.”

The participant differentiated between two types of privacy expectations: **expected desire** of how things should be and **expected thinking** of how things are. Furthermore, the participant suggested that we should clarify the type of privacy expectation.

“I think it is helpful if you make it clear otherwise you will get different answers and you don't know what they are answering to because some people might answer the question what they think it should be done, some people might answer the question as what it is, some people might not distinguish those […]”

The observation from the study suggested that people might have different types of privacy expectations and simply asking them what they expect could lead to different interpretations. We reviewed whether existing privacy literature treated privacy expectation as a multi-level construct, and we did not find that to be the case. Existing privacy theories considered privacy expectation as a single-level construct with one type. We summarize privacy literature related to privacy expectation in section 4.

### 3.2. Expectation-Related Theory in Non-privacy Literature

In contrast to the privacy domain, Consumer Satisfaction/Dissatisfaction (CS/D) and service quality domains treat expectation as a multi-level construct (Miller, 1977; Gilly et al., 1983; Zeithaml et al., 1993). CS/D literature supports four types of **consumer expectations**: *Ideal, Expected, Deserved* and *Minimum tolerable* (Miller, 1977; Gilly et al., 1983). Service quality literature supports three types of **service expectations**: *Desired, Adequate* and *Predicted* (Zeithaml et al., 1993). Consumer expectations and privacy expectations have conceptual similarities. Hence, we base our conceptual model for privacy expectation on the theories from the CS/D domain.

In the CS/D domain, consumer expectations are considered “an influence on, if not determinant of, levels of satisfaction or dissatisfaction.” (Gilly et al., 1983) Models of satisfaction consider two determinants of satisfaction: expected performance of a product and evaluation of its perceived actual performance (Miller, 1977). If the perceived actual performance is greater or equal to the expected performance consumers are satisfied otherwise dissatisfied. Miller extended this basic model of satisfaction to include **types** of expectations consumers might use as comparison standards for performance evaluation (Miller, 1977). As per Gilly et al.,

“Miller contends that simply asking the consumer what he or she ‘expects' can result in different interpretations by different consumers” (Gilly et al., 1983).

Miller conceptually recognized four types of consumer expectation types: *Ideal, Expected, Deserved*, and *Minimum Tolerable* (Miller, 1977). The *Ideal* represents a wished-for level that reflects what consumers feel performance **can be**. The *Expected* reflects what consumers think performance **will be**. It represents an objective calculation of probabilities and does not have an affective dimension. The *Deserved* has an affective dimension and represents what consumers feel performance **should be**. Lastly, the *Minimum Tolerable* represents what consumers feel the lowest performance **must be**. It is a better than nothing option.

Miller suggested an ordering among the types with Ideal at the highest level and Minimum Tolerable at the lowest. He contended that the Deserved would be higher than the Expected if consumer investment in terms of time, effort, money etc. is high. Empirical work by Gilly et al. found partial support for the types and ordering among them (Gilly et al., 1983).

Miller contends that consumer expectations probably vary among consumers based on experiences, demographics, psychographics etc., and they can vary within a consumer temporally based on recent experience, situation etc. (Miller, 1977). Privacy expectations can also vary among people based on demographic characteristics, privacy concern, privacy knowledge, geographic location etc, and they can vary within a person based on context, recent experience etc. (Rao et al., 2016).

## 4. Related Work

### 4.1. Theoretical Work on Privacy Expectation

Theoretical work on conceptualization of privacy expectation has largely considered it as a single-level construct (Altman, 1975; Nissenbaum, 2009; Lin et al., 2012; Martin, 2016) We discuss the conceptualizations below.

#### 4.1.1. Privacy as Boundary Regulation Process

Altman considers *desired privacy* and *achieved privacy* as two important aspects of privacy (Altman, 1975). He describes the desired level as a subjective ideal internal state at any given moment. If the achieved level of privacy, as perceived by an individual or group, matches the desired level, then satisfaction results, otherwise the individual or group is unsatisfied. Altman's work primarily focuses on physical interaction, and Palen and Dourish extend Altman's theory to a world with information technology (Palen and Dourish, 2003). They discuss how technology can disrupt privacy management by violating personal desires and social expectations of the social settings in which the technology is present.

#### 4.1.2. Privacy as Contextual Integrity

In Nissenbaum's *privacy as contextual integrity theory*, privacy expectations are obligatory norms (Nissenbaum, 2009, pp. 138–139), which vary by context and govern the flow of information in terms of who, what and how (Nissenbaum, 2009). Martin's *privacy as social contract theory* extends privacy as contextual integrity theory (Martin, 2016) and views privacy expectations as social contracts that are mutually beneficial, sustainable and unstated agreements within a context. In both the theories, meeting privacy expectations requires respecting obligatory norms.

#### 4.1.3. Privacy as Expectations

Lin et al. propose the concept of *privacy as expectations* (Lin et al., 2012). They study privacy expectations in a mobile context and define it as “people's mental models of what they think an app does and does not do.”

#### 4.1.4. Privacy Expectations as Likelihood

In our previous work, we proposed the concept of *privacy expectations as likelihood* (Rao et al., 2016). We used questions such as “What is the likelihood that [website name] would collect your information” to elicit expectations (Rao et al., 2016). We distinguished between two types of privacy expectations: expectations as likelihood and expectations as desires. We argued that in the privacy context most work had focused on expectations in the desired sense or preferences, or had not clarified the meaning of expectation. In this work, we build upon our prior work. We propose a conceptual model for privacy expectation with four distinct types and support the model with empirical evidence.

### 4.2. Empirical Studies on Privacy Expectation

Prior empirical studies related to privacy expectation have also considered privacy expectation as a single-level construct. In this work, we treat privacy expectation as a multi-level construct with four types and design an empirical study to measure them explicitly. Prior empirical studies can be classified into two categories.

The first category consists of studies that explicitly measure privacy expectation, but treat it as a single-level construct (Earp et al., 2005; Gomez et al., 2009; Milne and Bahl, 2010; Liu et al., 2011; Lin et al., 2012; Wijesekera et al., 2015; Martin and Shilton, 2016a,b; Micinski et al., 2017; Naeini et al., 2017; Senarath and Arachchilage, 2018). These studies explicitly specify the phrase “privacy expectation” in the study. While eliciting privacy expectations, they have largely focused on expectations in the desired sense (Milne and Bahl, 2010) or have not clarified the meaning of expectation (Earp et al., 2005; Gomez et al., 2009; Liu et al., 2011; Lin et al., 2012; Wijesekera et al., 2015; Martin and Shilton, 2016a,b; Naeini et al., 2017).

The second category consists of studies that do not explicitly measure privacy expectation. These studies do not explicitly specify “privacy expectation” in the study. Some of the studies elicit users' privacy preferences (Olson et al., 2005; Leon et al., 2013; Wang et al., 2016; Naeini et al., 2017). By measuring preferences, they may be implicitly measuring privacy expectation in the desired sense. Studies can also measure another privacy-related construct such as privacy concern (Smith et al., 1996; Phelps et al., 2000; Kumaraguru and Cranor, 2005), privacy attitude (Kumaraguru and Cranor, 2005) or privacy behavior (Pedersen, 1999; Berendt et al., 2005; Norberg et al., 2007), but not explicitly privacy expectation.

## 5. Conceptual Model of Privacy Expectation

We propose a conceptual model for *privacy expectation* inspired by the work on *consumer expectation* in Consumer Satisfaction/Dissatisfaction domain (Miller, 1977; Gilly et al., 1983) and *service expectation* in service quality domain (Zeithaml et al., 1993). Both domains treat expectation as a multi-level construct with different types. Each type of expectation is considered a *standard of evaluation*. For example, consumers can evaluate products by comparing actual product performance against Ideal, Expected, Deserved or Minimum Tolerable type of consumer expectation.

We propose a conceptual model for *privacy expectation* with four types: *Desired, Predicted, Deserved* and *Minimum*. By considering multiple types of privacy expectations, we treat privacy expectation as a multi-level construct. Each type of privacy expectation is a standard of evaluation against which people evaluate what they expect in a privacy context. When asked “What do you expect[…],” people can use any of these standards to evaluate what they expect in the scenario. Table 1 summarizes the four types of privacy expectations.

**Table 1:** Summary of conceptual model of privacy expectation types.

**Type**	**Keywords**	**Description**	**Critical^a^**	**Privacy level**
Desired	ideal, want	what people ideally **want** to happen		Highest
Predicted	think, will	what people **think** will happen	Knowledge	
Deserved	deserve, feel, should	what people **feel** should or ought to happen	Investment	
Minimum	tolerate, must	what people would **tolerate** if something must happen	Essentiality	Lowest

a *Critical determinant of Type, Knowledge of privacy practices and Investment in effort, time, money etc*.

*Bold values indicate important results discussed in the article*.

As discussed earlier, existing privacy theories generally consider privacy expectation as a single-level construct. Each privacy theory considers only one type of privacy expectation, which acts as a single standard of evaluation. However, there are differences in how each privacy theory conceptualizes privacy expectation. Below, we discuss the four proposed privacy expectation types and how they relate to existing conceptualizations of privacy expectation.

### 5.1. Desired Type

The *Desired* type is what people ideally want to happen. It is similar to the desired level of privacy used as the standard of evaluation in Altman's privacy theory (Altman, 1975). The desired level of privacy as per Altman is an ideal internal state at any moment, and people evaluate achieved level of privacy against the desired level of privacy.

### 5.2. Predicted Type

The *Predicted* type is what people think will happen. Here “will” indicates a definite future action or likely prediction. The Predicted type is similar to the *privacy as expectations* concept proposed by Lin et al. because their standard of evaluation is what people think a mobile app does or does not do (Lin et al., 2012). In our earlier work, we defined the concept of *privacy expectations as likelihood* as a measure of what users expect will likely happen (Rao et al., 2016). Hence, the Predicted type is also related to it. Accurately predicting website data practices may require knowledge of privacy practices. For example, a user who understands how IP address works may have different expectation about collection of location information than a user who does not. Our study on privacy expectations as likelihood found that a user's privacy knowledge impacted user expectations. For example, privacy knowledge impacted if a user expected the collection of health information in a particular scenario. Hence, we hypothesize that privacy knowledge will impact the Predicted type more than the other three types.

### 5.3. Deserved Type

Compared to other types, the Deserved type has an affective dimension that focuses on feelings. We consider that it is critically determined by evaluation of “investment and rewards” in a scenario. Therefore, the *Deserved* type is what people feel should or ought to happen given their investment. Here investment can be in terms of time, effort, money, loyalty etc. When investment is high, people can feel that they deserve a reward. For example, if people paid for a website service for a long time, they may feel that they deserve a reward such as 1 month of free service a year. On the contrary, when investment is low, people may feel that they do not deserve a reward. They may even feel that they deserve a penalty. For example, if they are not paying for a website service, they may feel they deserve to view unwanted advertisements, which could be perceived as a penalty. In a data economy, users can avail “free” services in exchange for their personal data, and companies can monetize the data via advertisements and other revenue models (Rao et al., 2016). Hence, in the privacy context, the penalty may be a decrease in privacy (Dinev and Hart, 2006).

The Deserved type is related to the standard of evaluation in Nissenbaum's *privacy as contextual integrity* theory and Martin's *privacy as social contract* theory. In Nissenbaum's theory, the standard of evaluation is based on context-relative informational norms (Nissenbaum, 2009). Nissenbaum considers norms that are obligatory (Nissenbaum, 2009, pp. 138–139). She attributes four key elements to norms including “[…] (a) a prescriptive “ought element”; (b) a norm subject upon whom the obligation expressed in the norm falls […].” Martin considers that individuals make decisions about sharing and use with obligations in mind (Martin, 2016). Because the Deserved type focuses on what people feel ought to happen, it is obligatory in the sense considered by Nissenbaum and Martin.

Martin considers that people use a rule-utilitarian approach that analyzes costs and benefits to develop norms (Martin, 2013). The cost may or may not be a decrease in privacy. Instead of “cost and benefit,” we prefer to use “investment and reward/penalty” in order to emphasize the affective dimension of the Deserved type. We hypothesize that investment and reward/penalty will impact the Deserved type more than other privacy expectation types.

### 5.4. Minimum Type

The *Minimum* type is what people would tolerate if something must happen; something is essential to fulfill a need and there is not much choice. Here “must” indicates a stronger obligation than “should” or “ought.” The Minimum type is critically determined by a lack of options from which people can choose based on desires or investment-reward analysis. For example, people may not generally tolerate collection of health information on a job website, but they may do so if it is required to apply for a specific job. The Minimum type is not strongly related to any standard of evaluation in existing privacy theories. Hence, it is our contribution to privacy theory.

### 5.5. Ordering of Privacy Expectation Types

We hypothesize that there can be an ordering among the privacy expectation types. Different types of privacy expectations can represent different levels of user privacy. If people were to assign a score to each type, there could be an ordering among the scores. Given that the Desired type is the most ideal type, it would have the highest score. In contrast, the Minimum type is something that is just tolerated, and, hence, would have the lowest score. We hypothesize that the scores for Predicted and Deserved would be between the scores for Desired and Minimum. The Deserved score could be higher than the Predicted score if “investment” is high, otherwise its score would be lower than the Predicted score.

## 6. Empirical Study

We designed an empirical study to test our proposed conceptual model for privacy expectation types. We use the study to provide evidence that contradicts the prevailing assumption that privacy expectation is a single-level construct. It supports our claim that privacy expectation is a multi-level construct. The empirical study tested the following hypotheses:

- Are there statistically significant differences among Desired, Predicted, Deserved and Minimum privacy expectation types?
- Is there a statistically significant difference between the orderings *Desired*>*Predicted*>*Deserved*>*Minimum* and *Desired*>*Deserved*>*Predicted*>*Minimum*?
- Is the impact of knowledge significantly more on the Predicted type?
- Is the impact of investment significantly more on the Deserved type?

We use a contradictory evidence approach. In this approach, a single piece of evidence that contradicts the existing assumption is sufficient. For instance, existence of a single black swan is sufficient to contradict the assumption that all swans are white. We use results from a single realistic scenario as contradictory evidence. To make the evidence stronger, we selected an extreme or deviant case scenario that was unlikely to support a multi-level conceptualization of privacy expectation.

### 6.1. Sample and Procedure

We conducted the study as per the institutional guidelines for human-subject research and adhered to the basic principles of ethical research. We ran the study in August 2017 with an initial sample consisting of 1,437 adults (18+ years) selected from a United States online survey panel (SurveyMonkey, 2019). The sample consists of US adults with access to the Internet and is age and gender balanced as per US census. It is a stratified random sample, which reduces self-selection bias. A total of 1249 participants completed the survey (completion rate 86.91%). The final sample consisting of participants who completed the survey (*N*=1,249) is representative of the US online population with a margin of error of ±2.8%.

Participants selected from the online survey panel were invited to take a self-administered questionnaire that elicited their privacy expectations regarding a realistic privacy-sensitive scenario involving collection of health-related browsing activity by a bank. We received informed consent at the beginning of the survey. Participants completed the survey to donate $.50 to their preferred charity and enter a sweepstakes to win a $100 gift card (odds of winning 1/60,000). On average, panelists can take two surveys per week (SurveyMonkey, 2019). There were no repeat participants in our survey, which reduced the impact of learning effects.

### 6.2. Variables

The independent variable in our study is the privacy expectation type with four levels: Desired, Predicted, Deserved and Minimum. The dependent variable is the participant's privacy expectation rating for a given expectation type. Participants expressed their privacy expectations by rating their level of agreement or disagreement to four statements corresponding to the four expectation types. We used a within-subjects (repeated-measures) design where each participant rated all expectation types. We manipulated the independent variable by varying the description of four statements. To reduce order effects, we reversed the expectation questions for half of the participants.

### 6.3. Study Scenario

As discussed earlier, we use a contradictory evidence approach. Although privacy is contextual and expectations may vary in different scenarios, evidence from one ecologically valid scenario is sufficient to support our main hypothesis: “do distinct types of privacy expectations exist?” To this end, we choose a realistic scenario. Furthermore, to provide strong evidence for our main hypothesis, we use an extreme or deviant case scenario where our main hypothesis is likely to fail. Generally, differences found in an extreme or deviant case scenario are likely to be amplified in an average case scenario.

To ensure that the results from the study are meaningful, we chose a scenario based on reality. Banks in the United States can collect health information from users (Rao et al., 2016; PNC Bank, 2017; Bank of America, 2019). For instance, Bank of America, the second largest commercial bank in the United States (The Federal Reserve, 2019) collects protected health information from users (Rao et al., 2016; Bank of America, 2019). Banks can collect health-related browsing activities of users from health websites using tracking mechanisms (Rao et al., 2016; PNC Bank, 2017; Bank of America, 2019). For example, PNC Bank, the sixth largest commercial bank in the United States (The Federal Reserve, 2019) can collect health-related browsing activities of users (PNC Bank, 2017). Ghostery (ghostery.com), a tool to identify trackers showed several trackers on popular health websites such as WebMD (71), MedlinePlus (4), and MedicineNet (16). Tracking mechanisms can allow banks to uniquely identify users e.g., by name and postal address (Rao et al., 2014).

The study elicited users' privacy expectations regarding a realistic scenario involving collection of health-related browsing activity by a bank. In addition to being realistic, this was an extreme or deviant case scenario that was unlikely to support our main hypothesis, and the four privacy expectation types would not be significantly different. For example, given the sensitive nature of health-related information, it was unlikely that users would desire collection of health-related information by banks (Desired expectation). Our prior study showed that people did not predict collection of health-related information by banks (Rao et al., 2016). Hence, users were unlikely to predict collection of health-related information by banks (Predicted expectation). Therefore, Desired and Predicted expectations would not be different.

To elicit privacy expectations regarding a data practice, empirical studies decompose the data practice into five components: *action, data, source, target* and *purpose* (Lin et al., 2012; Martin and Shilton, 2016a,b). We decomposed the banking data practice as follows. The *action* was *collection*. The *data* was *health-related browsing activity*. We defined it as browsing activities on websites such as WebMD, MedlinePlus or MedicineNet that people might use/visit to find information on health conditions, symptoms or treatments. The *source* of the data was the *participant*, and the *target* was a *bank*. The *purpose* of data collection was specified as “*to identify financial needs and provide relevant service*.” To ensure realism, the wording was based on the privacy policy of PNC Bank (PNC Bank, 2017), the sixth largest commercial bank in the United States (The Federal Reserve, 2019).

### 6.4. Questionnaire Design

As per survey best practices, the survey wording was iteratively improved based on the feedback from Cognitive Interviews (Willis, 2004) and pilot studies. During the Cognitive Interviews (*N* = 5), we asked the participants to the express in their own words what they understood from each question. We wanted to ensure that the wording conveyed what we wanted to measure. At the end of the first (*N* = 130) and the second (*N* = 60) pilot studies, we asked the participants if they had difficulty answering any question, and if yes, what about the question made it difficult to answer. Participants were also given the option of suggesting improvements to the questions. Overall feedback suggested that the target group interpreted the questions in the way we intended and was able to understand the differences among the four privacy expectation types. The complete survey questionnaire is available in the Supplementary Material.

We did not use attention check questions as per advice from recent research on survey methodology (Berinsky et al., 2014; Clifford and Jerit, 2015; Anduiza and Galais, 2017). Such questions may increase Social Desirability Bias (Clifford and Jerit, 2015), which is an important issue for surveys related to privacy. Discarding responses based on attention check questions can introduce demographic bias related to gender, age and education (Berinsky et al., 2014; Clifford and Jerit, 2015; Anduiza and Galais, 2017), which can impact nationally representative surveys. Lastly, our pilot results indicated that the median completion time was short ~3min, which reduces the decline in attention due to satisfying behavior.

#### 6.4.1. Survey Introduction

To reduce the impact of demand characteristics, we informed the participants that the purpose of the survey was to understand their opinions regarding websites. Asking for opinions also reduces the threat of knowledge questions and decreases guessing (Bradburn et al., 2004, pp. 203-205). To further reduce guessing, we told them that they should answer the questions as accurately as possible, but it was OK to say “Don't know.” We did not mention “privacy” to avoid priming effects. To reduce social desirability bias, participants were assured that their answers were anonymous, and that we did not collect any personally identifiable information including IP address. We told the participants that their answers were important to us, and they could take their time reading and answering the questions.

#### 6.4.2. Pre-questionnaire

To provide context regarding our study scenario, we asked participants about their usage of health-information and banking websites. We also measured their “investment” to test the hypothesis whether investment impacted the Deserved type significantly more than other types.

To reduce order effects, the two blocks related to health (2 questions) and banking (3 questions) were shown in random order. Questions were worded to reduce social desirability bias e.g., “Some people use […] Other people do not[…].” Answer options included “Don't know/ Not sure” and “Decline to answer.”

The health block had two closed-ended questions. First asked whether participants had used websites such as WebMD, MedlinePlus or MedicineNet to find information on health conditions, symptoms or treatments. Second asked them to think about their last visit and tell us whether they recalled the information they were trying to find.

The banking block had three closed-ended questions. First question asked if participants had used websites to check Checking/Savings account balance. Second question asked whether they currently had a Checking/Savings account. Third question asked the approximate year in which they opened their account. We considered the number of years since opening the account as a measure of “investment” that could impact the Deserved type.

#### 6.4.3. Main Questionnaire

We instructed the participants to imagine a scenario where they were a customer of a bank, and they had a Checking/ Savings account with the bank. Each participant rated four Likert-type items one each for Desired, Predicted, Deserved and Minimum privacy expectation types. Ratings were used as the dependent variable to analyze the impact of the independent variable, privacy expectation type. For the rating task, participants were instructed.

“In this scenario, tell us how much you **agree or disagree** with the statements below. Use a scale from 0 to 10, with 0 indicating strongly disagree and 10 indicating strongly agree.”

To distinguish neutral from undecided, the scale included a “Don't know/ Not sure” option. Explicitly including a “Don't know” option is important for determining level of knowledge (Bradburn et al., 2004, pp. 203–205). A “Don't know” option reduces guessing and indicates that “Don't know” answers are expected and acceptable (Bradburn et al., 2004, pp. 203–205). We hypothesized that knowledge impacts the Predicted type significantly more than the other types, and a “Don't know” option allowed us to test that hypothesis. Because of the privacy-sensitive scenario, the scale also included a “Decline to answer” option.

Prior empirical studies (Martin and Shilton, 2016a,b) have used a similar rating scale to measure and compare user privacy expectations for multiple items. By measuring level of agreement, we can compare users' rating for four expectation-related items. An 11-point scale measures finer differences among four expectation types. It allows participants to distinguish among four items with a probability (~54%) greater than chance (50%). Smaller scales have a probability less than chance e.g., 9-point (~46%), 7-point (~35%) and 5-point (~19%). Likert-type item data measured on a 11-point scale is closer to interval level of scaling (Leung, 2011), which can be used with more powerful statistical tests.

We empirically elicited four types of privacy expectations similar to how Gilly et al. empirically elicited four types of consumer expectations (Gilly et al., 1983). Participants rated how much they **agree or disagree** with the four statements given below. The statements used keywords identified in the conceptual model (Table 1) to capture the impact of four privacy expectation types: **want** for Desired, **think…will** for Predicted, **deserve** for Deserved and **tolerate…must** for Minimum.

“I **want** my bank to collect my health-related browsing activity to identify my financial needs and provide service relevant to me.”

“I **think** my bank **will** collect my health-related browsing activity to identify my financial needs and provide service relevant to me.”

“I **deserve** that my bank collect my health-related browsing activity to identify my financial needs and provide service relevant to me.”

“I would **tolerate** if my bank **must** collect my health-related browsing activity to identify my financial needs and provide service relevant to me.”

Stating both sides of the attitude scale (**agree or disagree**) and a repeated-measures design reduced the impact of acquiescence bias. Each statement was a positive affirmative statement without double-negatives. It ensured that higher scores corresponded to higher levels of agreement to collection of data. We stated the purpose of collection as “to identify my financial needs and provide service relevant to me.” As discussed earlier, the purpose was carefully chosen to ensure realism. Instructions for the rating task provided a definition for “health-related browsing activity.”

#### 6.4.4. Post-questionnaire

We asked demographic questions at the end of the survey. We asked a question regarding the highest education level completed. We received information about gender (male, female), age range (18–29, 30–44, 45–59, 60+), household income and US location for each participant from the survey panel. We asked two open-ended questions soliciting participant feedback. They could explain if they had difficulty in answering questions and tell us about issues that they thought were important for the study.

## 7. Results

We analyzed all completed survey responses (*N* = 1, 249). The median time to complete the survey was 3 min and 8 s. Table 2 lists participant demographics gender, age range, highest education level, household income, and US geographical region. For analysis, we set the level of significance α = 0.05. We adjusted α for pairwise comparisons using Bonferroni correction. For example, we set α = 0.008 for comparisons between four expectation types.

**Table 2:** Participant demographics (*N* = 1, 249).

**Gender**	***N***	**%**
**Female**	672	53.80%
**Male**	577	46.20%
**Age range (years)**	*N*	%
**18–29**	231	18.49%
**30–44**	338	27.06%
**45–59**	229	18.33%
**60+**	451	36.11%
**Education**	*N*	%
**Grade 1-8/ no formal school**	5	0.40%
**Grade 9-11/ 12 no diploma**	29	2.32%
**Grade 12 with diploma**	128	10.25%
**Some college, no degree**	256	20.50%
**Two-year college degree**	113	9.05%
**Four-year college degree**	308	24.66%
**Some postgraduate school**	102	8.17%
**Postgraduate degree**	291	23.30%
**Decline to answer**	17	1.36%
**Household income**	*N*	%
**$0 to $9,999**	92	7.37%
**$10,000 to $24,999**	133	10.65%
**$25,000 to $49,999**	231	18.49%
**$50,000 to $74,999**	164	13.13%
**$75,000 to $99,999**	137	10.97%
**$100,000 to $124,999**	120	9.61%
**$125,000 to $149,999**	59	4.72%
**$150,000 to $174,999**	32	2.56%
**$175,000 to $199,999**	26	2.08%
**$200,000 and up**	61	4.88%
**Decline to answer**	194	15.53%
**US region**	*N*	%
**East North Central**	186	14.89%
**East South Central**	63	5.04%
**Middle Atlantic**	151	12.09%
**Mountain**	111	8.89%
**New England**	71	5.68%
**Pacific**	222	17.77%
**South Atlantic**	226	18.09%
**West North Central**	98	7.85%
**West South Central**	109	8.73%
**Decline to answer**	12	0.96%

### 7.1. Multiple Privacy Expectation Types Exist

Empirical results support the hypothesis that privacy expectation is a multi-level construct. People can have multiple types of privacy expectations.

#### 7.1.1. Privacy Expectation Types

To examine participants' ratings for the four expectation types, we considered numerical (0-strongly disagree to 10-strongly agree) (*n* = 1, 038), but not “Don't know” or “Decline to answer” responses. We treated the ratings, measured on a fine grained 11-point scale, as interval data. Table 3 lists mean, SD, minimum, quantiles and maximum of the ratings for four expectation types and their six pairwise comparisons. The median ratings for all expectation types are 0. At least 50% of the participants strongly disagreed to collection of health-related browsing activity by a bank; these participants did not desire it (Desired), predict it will happen (Predicted), feel they deserved it (Deserved), or tolerate it under any circumstances (Minimum). Ratings for Desired and Deserved are 0 even at the 75*th* quantile. However, at the 75*th* quantile, ratings for Predicted and Minimum are ≥1 indicating that participants disagree to a lesser extent. At the 90*th* quantile, about 10% of the participants somewhat agree (≥5) that banks will collect health-related browsing activity data, and they tolerate such collection under some circumstances. Even at the 90*th* quantile, participants disagree (≥3) that they desire or deserve such collection.

**Table 3:** Participant ratings of privacy expectation types (*n* = 1,038).

**Type**	**Mean**	***SD***	**Min**	***Q***	***Q***	***Q***	***Q***	***Q***	**Med**	***Q***	***Q***	***Q***	***Q***	***Q***	**Max**
				**5%**	**10%**	**15%**	**20%**	**25%**		**75%**	**80%**	**85%**	**90%**	**95%**	
Predicted	1.48	2.58	0	0	0	0	0	0	0	2	3	5	6	8	10
Minimum	1.13	2.36	0	0	0	0	0	0	0	1	2	3	5	7	10
Deserved	0.91	2.15	0	0	0	0	0	0	0	0	1	2	3.1	6	10
Desired	0.84	2.12	0	0	0	0	0	0	0	0	1	2	3	6	10
P-Di	0.64	2.14	-10	-1	0	0	0	0	0	0	1	2	3	5	10
P-De	0.58	2.17	-10	-1	0	0	0	0	0	0	0	1	3	5	10
P-M	0.35	2.45	-10	-3	-1	0	0	0	0	0	0.2	1	3	5	10
M-Di	0.29	1.69	-10	-1	0	0	0	0	0	0	0	1	2	3	10
M-De	0.22	1.54	-10	-1	0	0	0	0	0	0	0	0	1	2	10
De-Di	0.07	1.29	-10	-1	0	0	0	0	0	0	0	0	0	1	10

*The gray shades indicate important results discussed in the article*.

As seen from Table 3, the mean values of the ratings for the four expectation types are different: Predicted (1.48), Minimum (1.13), Deserved (0.91), and Desired (0.84). However, the distribution of ratings is not normal, and mean values may not accurately estimate significance. Hence, we compare rank ordering of the ratings by using nonparametric tests that treat rankings as ordinal data. Friedman test for measuring differences between related observations (Conover, 1999), indicated a significant overall difference among the expectation types *F*_(3)_ = 53.4264; *p* < 0.00001. Pairwise comparisons of expectation types showed significant differences *p* < 0.00003 within all pairs except one: (Desired, Deserved). The pairs (Predicted, Desired), (Predicted, Deserved), (Predicted, Minimum), (Minimum, Desired), and (Minimum, Deserved) were significantly different. This result supports the hypothesis that people have different types of privacy expectations.

The overall difference between Desired and Deserved types is not significant. There is strong positive correlation (Spearman ρ = 0.81, *p* < 0.0001) between them. This indicates that people who have higher desire for collection of health information feel more strongly that they deserve such collection. Results may be different in other scenarios. In a less privacy-sensitive scenario, we may find negative correlation. For example, when watching movies for free, people may have low desire for ads, but feel strongly that they deserve ads. Interaction between context sensitivity and the Deserved type needs further investigation.

#### 7.1.2. Population Estimates

Using Binomial interval estimation, we computed interval estimates for *p*, the proportion of the population that would give different scores for two different privacy expectation types. We assume that our sample (*n* = 1, 038) is representative of the population. We computed 99.2% confidence intervals for pairs of privacy expectation types using the Wilson score. Between 19% to 26% of the population rates Desired and Minimum differently indicating that, even in a privacy-sensitive scenario, the desired level of privacy can differ from the minimum tolerable level of privacy. The largest proportion of the population, 29% to 36%, rates Predicted and Minimum differently. The smallest proportion, 13% to 19%, rates Deserved and Desired differently.

### 7.2. Privacy Expectation Types Can Be Ordered

We hypothesized that there may be a statistically significant difference between the two orderings of expectation types *Desired* > *Predicted* > *Deserved* > *Minimum* and *Desired* > *Deserved* > *Predicted* > *Minimum*. In our study, higher ratings indicate higher agreement to collection of health-related browsing activities. Hence, higher ratings indicate lower privacy expectations. Therefore, to test the significance of ordering, we analyze the reversed versions *Desired* < *Deserved* < *Predicted* < *Minimum* and *Desired* < *Predicted* < *Deserved* < *Minimum*. Page test for ordered alternatives (Conover, 1999) is significant for *Desired* < *Deserved* < *Predicted* < *Minimum* (*T* = 5.37; *p* < 0.001), but not for *Desired* < *Predicted* < *Deserved* < *Minimum* (*T* = 1.77; *p* < 0.039) at Bonferroni adjusted α = 0.025. This supports our hypothesis that privacy expectation types can be ordered. The Desired level of privacy is higher than the Minimum level of privacy. This indicates that a person can ideally wish for a higher level of privacy and yet tolerate a lower level of privacy when essential.

### 7.3. Knowledge Impacts the Predicted Type

We hypothesized that knowledge may impact the Predicted type more than the other types. The proportion of “Don't know/ Not sure” responses is significantly higher for the Predicted type. People seem to believe that they need knowledge to express their Predicted privacy expectation. Hence, knowledge does impact the Predicted type more than the other types.

We analyzed the frequencies of “Know (0–10)," “Don't know/Not sure” and “Decline to answer” responses for the four expectation types (Table 4). The number of responses that contain “Decline to answer” is small and similar among the four expectation types (20, 1.60%; 21, 1.68%; 26, 2.08%; 20, 1.60%). However, the number of “Don't know” responses is higher for Predicted (151, 12.09%) compared to Desired (52, 4.16%), Deserved (67, 5.36%) and Minimum (49, 3.92%) expectation types. We removed “Decline to answer” responses from our analysis.

**Table 4:** Responses for privacy expectation types (*N* = 1, 249).

**Type**	**Know (0–10)**	**Don't know**	**Decline to answer**
Desired	1,177 (94.24%)	52 (4.16%)	20 (1.60%)
Predicted	**1,077 (86.23%)**	**151 (12.09%)**	21 (1.68%)
Deserved	1,156 (92.55%)	67 (5.36%)	26 (2.08%)
Minimum	1,180 (94.48%)	49 (3.92%)	20 (1.60%)

*Bold values indicate important results discussed in the article*.

We compared frequencies of “Don't know” with “Know” responses for the four expectation types by considering the responses as dichotomous nominal values. Cochran's Q test for related observations (Conover, 1999) indicated a significant overall difference in the frequency of “Don't know” responses among the expectation types *Q*_(3)_ = 177.27; *p* < 0.001. Pairwise comparisons between expectation types indicated that the frequency of “Don't know” responses was significantly different for the Predicted expectation type compared to Desired *Q*(1) = 80.03; *p* < 0.001, Deserved *Q*_(1)_ = 59.11; *p* < 0.001 and Minimum *Q*_(1)_ = 87.19; *p* < 0.001 types. This supports the hypothesis that knowledge impacts the Predicted type more than other types.

### 7.4. Investment Impacts the Desired Type

We hypothesized that evaluation of “investment and reward” in a scenario would critically impact the Deserved type. We used the duration that participants have had their banking account as an indication of their investment. Results partially support this claim. Investment impacts whether people feel that they deserve a data privacy practice or not.

Spearman pairwise rank correlations showed significant (*p* < 0.0001) weak negative correlations between investment and ratings of all expectation types: Minimum (ρ = −0.20), Deserved (ρ = −0.19), Desired (ρ = −0.19) and Predicted (ρ = −0.18). However, investment has significant moderate correlation with age (ρ = 0.54). To control for the impact of age, we analyzed the impact of investment on each age group.

In the 18–29 age group, there is significant weak to moderate negative correlation (ρ = −0.28, *p* = 0.0016) between investment and the Deserved type. However, there is no significant correlation between investment and other expectation types. Among other age groups, 30–44, 45–59, and 60+, there is no significant correlation between investment and any expectation type. This implies that only in the 18–29 age range, low investment significantly increases the feeling that one deserves collection of data, and high investment significantly decreases the feeling that one deserves collection of data. This partially supports the hypothesis that investment impacts the Deserved type more than other types.

### 7.5. Privacy Expectation Types Vary by Groups

Analysis (*n* = 1, 038) shows that people in different groups may rate a privacy expectation type differently. We analyzed groups based on demographics (gender, age range, household income and education level) and experience (prior use of websites and duration of bank account). Based on the number of levels of demographic and experience attributes, we set Bonferroni adjusted α = 0.002. For pairwise comparisons, α value was further divided by the number of comparisons. There was no significant difference in scores based on prior use of health and banking websites.

#### 7.5.1. Gender

The survey panel categorized participants into male or female categories. Gender does not seem to influence expectations about data privacy practices. Mann-Whitney test found no significant differences in scores by gender for the four privacy expectation types.

#### 7.5.2. Age

The study categorized participants into four age ranges: 18–29, 30–44, 45–59, and 60+. Results show that age influences expectations about data privacy practices. Kruskal-Wallis Rank Sum test indicated significant overall difference in scores by age range: Minimum χ2_(3)_ = 83.40, *p* < 0.0001; Deserved χ2_(3)_ = 49.64, *p* < 0.0001; Desired χ2_(3)_ = 45.68, *p* < 0.0001; and Predicted χ2_(3)_ = 36.72, *p* < 0.0001. Hence, the four age groups have differences in privacy expectation types. Spearman pairwise rank correlations showed significant (*p* < 0.0001) weak negative correlations between age range and scores of all expectation types: Minimum (ρ = −0.25), Deserved (ρ = −0.21), Desired (ρ = −0.19), and Predicted (ρ = −0.19). This indicates that as age increases, people agree less to collection of health-related browsing activities.

Younger people desire, predict, feel that they deserve, and tolerate data privacy practices differently than older people. Pairwise comparisons using Wilcoxon test showed significant differences by expectation type and age range. For the Minimum type, expectations were different (*p* < 0.0001) between 18–29 vs. 30–44, 18–29 vs. 45–59, and 18–29 vs. 60+. The minimum tolerable expectations of the 18–29 age range is significantly different compared to all other age ranges. Both for the Deserved type and the Desired type, expectations were different (*p* < 0.0003) between 18–29 vs. 45–59, 18–29 vs. 60+ and 30–44 vs. 60+. The desired and deserved expectations of 18–29 year old were not significantly different than 30–44 year old. Lastly, for the Predicted type, expectations were different (*p* < 0.0001) between 18–29 vs. 60+ and 30–44 vs. 60+. Note that 18–29 and 60+ age ranges have significant differences in all types of privacy expectations.

#### 7.5.3. Education Level

Spearman pairwise rank correlations showed significant (*p* < 0.0003) weak negative correlations between education level and Minimum (ρ = −0.13), Deserved (ρ = −0.12), Desired (ρ = −0.11) types. Hence, people with lower level of education seem to have a stronger desire and higher tolerance for collection of health data than people with higher level of education. They also feel more strongly that they deserve such collection. There was no significant correlation between education level and the Predicted type. Hence, level of education does not seem to impact whether people predict such collection.

#### 7.5.4. Household Income

The impact of household income is similar to that of education level. Spearman pairwise rank correlations showed significant (*p* < 0.00014) weak negative correlations between household income and Minimum (ρ = −0.23), Deserved (ρ = −0.21), Desired (ρ = −0.19) types, but not the Predicted type. Hence, household income impacts how much people desire, tolerate and feel that they deserve collection of health data. However, it does not seem to impact whether people predict such collection.

## 8. Limitations

In this section, we discuss the limitations of our work. We discuss in particular threats to validity and summarize how we mitigated them in our study.

### 8.1. Construct Validity

We used Cognitive Interviewing process (Willis, 2004) to ensure that the study questionnaire measured what we intended it to measure. The questionnaire wording was iteratively improved based on the feedback from Cognitive Interviews (*N* = 5). We also improved the wording based on open-ended feedback from participants in two pilot studies (*N* = 130 and *N* = 60). Overall feedback indicated that the target group was able to understand the questionnaire and differentiate among the four privacy expectation types. This reduced the threat to construct validity.

As with all models, the proposed conceptual model for privacy expectation types may neither be exhaustive nor complete. The listed types may not exhaustively capture all possible types. Furthermore, the model may not completely characterize all the types represented. However, five decades of research on expectation types in non-privacy domains has identified a maximum of four types, and existing privacy theory has identified only three types. This gives a certain level of confidence regarding the number of possible privacy expectation types. Nevertheless future research may discover other types.

### 8.2. Ecological Validity

To ensure meaningful results, we selected a scenario based on reality. Top 10 banks in the United States, e.g., Bank of America and PNC Bank, collect health information from users (Rao et al., 2016; PNC Bank, 2017; Bank of America, 2019). However, users are predominantly not aware of such practices (Rao et al., 2016). We conducted an online study to elicit user expectations. Although, the study captures how users generally interact with online services, it may be beneficial to supplement the study with in-lab studies conducted under more controlled conditions.

### 8.3. Internal Validity

We followed several best practices in our study design to address internal validity of the results. We used a within-subjects (repeated-measures) design to remove variance due to differences between subjects and address factors such as participant stress and background that are not easy to control. Following guidelines from related studies (Gilly et al., 1983; Martin and Shilton, 2016a,b), we used partial counterbalancing to control for order effects and maximize experimental power. A larger sample size and complete counterbalancing could further control for order effects without reducing experimental power.

In section 6, we discuss how we address learning effects, completion rate, impact of demand characteristics, priming effects, social desirability bias, threat of knowledge questions and acquiescence bias. To address history threat, it would be beneficial to conduct further longitudinal studies.

### 8.4. External Validity

Our sample (*N* = 1,249) consists of United States adults with access to the Internet. The stratified random sample is balanced on age and gender and is representative of the US online population (±2.8%). A stratified random sample reduces self-selection bias. Although the sample is representative of an online population, it is not necessarily representative of the general population. Furthermore, the results from the sample may not be generalizable to other countries. It would be interesting to conduct further studies on samples from other countries and compare them with our results.

Our empirical study elicited users' privacy expectations in a single, extreme-case, privacy-sensitive scenario. The choice of scenario supported the main goal of the study, which was to provide evidence for the existence of multiple types of privacy expectations. Generalizability of results, although an important endeavor, was not the primary focus of the study. The more important focus was transferability, and researchers can use the conceptual model as a guideline for designing their studies related to privacy expectation types.

As part of future work, it would be useful to study privacy expectation types in other scenarios. For example, in a less privacy-sensitive scenario, we hypothesize that there may be larger differences between users' Desired expectation and Minimum expectation, but their Desired expectation may be more aligned with their Predicted expectation.

### 8.5. Reliability

Results from our main study (*N* = 1, 249) and two pilot studies (*N* = 130 and *N* = 60) are similar suggesting that results are repeatable. Further studies can provide higher degree of confidence in reliability.

## 9. Implications and Conclusions

We proposed a conceptual model of privacy expectation with Desired, Deserved, Predicted, and Minimum types, and ordering among the types. Empirical results supported the conceptual model and showed that people have distinct types of privacy expectations. We tested the proposed conceptual model in an extreme or deviant case where existence of distinct types of privacy expectations was unlikely. However, we found significant differences among them even in an extreme case. Furthermore, we found this result repeatable under two different user samples and generalizable to the US population with access to the Internet.

As we note in the limitations section, we do not make broad claims related to generalizability of the proposed conceptual model for privacy expectation types. Although generalizability of results is important, our focus is on a related concept: transferability. While generalizability focuses on the applicability of results from one scenario in a different scenario, transferability focuses on the process of other researchers transferring our results to their own studies. We believe that the conceptual model of privacy expectation types is transferable, and researchers can use the model as a guideline for designing their studies related to privacy expectation types. Because we found support for the model in an extreme case, it is advisable that researchers account for different expectation types in their design. If not, researchers may measure privacy expectation incorrectly.

We hope our results will foster more research into privacy expectation types, and the proposed conceptual model can provide coherent guidance for future studies related to privacy expectations. Below, we conclude with important implications.

### 9.1. Studies Must Explicitly Consider the Type of Privacy Expectation

To measure privacy expectations accurately, studies must identify and elicit the type relevant for the study. Studies in the past have inadvertently not done so (Earp et al., 2005; Gomez et al., 2009; Liu et al., 2011; Lin et al., 2012; Wijesekera et al., 2015; Martin and Shilton, 2016a,b). For example, one study used “would you **expect** it to access your precise location?” to measure what users think i.e., elicit expectations related to the Predicted type (Lin et al., 2012). Another study used “how much did you **expect** this app to be accessing this resource?” to measure what users desire i.e., elicit expectations related to the Desired type (Wijesekera et al., 2015). Two studies asked users to rate the statement “This application meets my **privacy expectations**” to elicit expectations related to the Deserved type (Martin and Shilton, 2016a,b). To measure expectations accurately, these studies must make the privacy expectation type explicit. The design of our empirical study can inform the design of future studies.

### 9.2. Privacy Research Must Focus on All Privacy Expectation Types

By largely studying user preferences, research in the privacy domain has implicitly focused on the Desired type. It is important to study other types of privacy expectations. For instance, Turow et al. found that 66% of Americans do not desire websites to show them ads tailored to their interests (Turow et al., 2009). However, Americans' Desired level is different than their Minimum level even in a privacy sensitive-scenario. Hence, it is important to study whether tailored ads meet Americans' minimum expectations.

Studies that measure the privacy paradox (Berendt et al., 2005; Norberg et al., 2007) i.e., the gap between stated privacy preferences and actual privacy behavior may benefit from considering the Minimum expectation type in addition to preferences or the Desired type. If the studies also measured the Minimum expectation, they may find that the gap is smaller and the stated behavior is more aligned with the actual behavior.

Research in the Consumer Satisfaction/ Dissatisfaction (CS/D) domain shows that the gap between expected performance, based on a type, and perceived actual performance can significantly predict satisfaction. In the privacy domain, gap between expected privacy, based on a type, and perceived actual privacy may predict both satisfaction and privacy concerns. For example, the gap between the Desired level and reality may better predict satisfaction than the gap between the Minimum level and reality. People may be better satisfied when their desires more closely match reality than when minimum tolerable expectations match reality. Conversely, the gap between the Minimum level and reality may better predict privacy concern than the gap between the Desired level and reality. People may be more concerned when even minimum tolerable expectations do not match reality.

### 9.3. Studying Privacy Expectation Types Can Benefit Regulators

Consumer privacy protection agencies (Council of The European Union, 2016; The U.S. Federal Trade Commission, 2019) can identify scenarios that have a stronger need for intervention e.g., scenarios where data practices fail to meet even Minimum privacy expectations. Studying multiple expectation types provides a more nuanced view of users' privacy expectations. Regulators can identify whether users desire, understand, feel that they deserve or tolerate data privacy practices.

## Data Availability Statement

The dataset is publicly available in the https://doi.org/10.7910/DVN/WJQAXT (Rao and Pfeffer, 2020).

## Ethics Statement

Ethical review and approval was not required for the study on human participants in accordance with the local legislation and institutional requirements. Basic principles of ethical research were followed in conducting this study. The participants provided their written informed consent to participate in the study.

## Author Contributions

AR and JP designed the study and wrote the manuscript. AR conducted the study and analyzed the result.

### Conflict of Interest

The authors declare that the research was conducted in the absence of any commercial or financial relationships that could be construed as a potential conflict of interest.
