# Supplementary material for: Types of Privacy Expectations
Source: Front Big Data. 2020 Feb 25;3:7. doi: 10.3389/fdata.2020.00007 (PMC7931868; doi:10.3389/fdata.2020.00007)
Supplement: Supplementary file 1 [file Data_Sheet_1.PDF]

[Informed Consent]

This is a research study conducted by an academic university. The purpose of this study is to understand your opinions regarding websites. You must be 18 years or older to participate, and your participation is voluntary.

As part of the study, you will take a survey that will last about 10 minutes. It contains questions regarding your opinions and usage of websites, and your background.

Your answers are anonymous. **We will NOT collect personal information, IP address or other personally-identifiable information**. We receive the following demographic information from SurveyMonkey: age range, gender, type of device, annual household income and region. The risks associated with participation in this study include boredom and fatigue, and are not greater than those ordinarily encountered in daily life or online activities.

Do you understand the information above, are 18 years or older, and want to continue with the survey?
☐ Yes, I understand the information above, am 18 years or older, and want to continue with the survey
☐ No, do not continue with the survey

[Survey Instructions]

Thank you for your interest in our survey. Your answers are important to us.

Please read the following instructions carefully:

Take your time in reading and answering the questions.
Answer the questions as accurately as possible.
It is OK to say that you don't know an answer.

[Pre questionnaire]
In this survey, we would like to understand your opinions regarding websites.

First, we will ask a few questions about websites that you might use.

[To minimize order effect, randomize the order of health and banking questions]

Some people use websites such as WebMD, MedlinePlus or MedicineNet to find information on health conditions, symptoms or treatments. Other people do not do so.
As far as you can recall, have you ever used websites such as WebMD, MedlinePlus or MedicineNet to find information on health conditions, symptoms or treatments?
() Yes
() No
() Don't know/Not sure
() Decline to answer

[Omit next question if not answered "Yes" to the previous question]

Please think about the last time you used a website such as WebMD, MedlinePlus or MedicineNet to find information on health conditions, symptoms or treatments. Try to recall the information that you were trying to find.
Do you recall what information you were trying to find?
() Yes
() No
() Don't know/Not sure
() Decline to answer

Some people use websites to check their Checking/Savings account balance. Other people do not do so.
As far as you can recall, have you ever used a website to check your Checking/Savings account balance?
() Yes
() No
() Don't know/Not sure
() Decline to answer

Some people have a Checking/Savings account. Other people do not.
Do you currently have a Checking/Savings account?
() Yes
() No
() Don't know/Not sure
() Decline to answer

[Omit next question if not answered "Yes" to the previous question]

To understand whether opinions regarding banking websites vary based on the length of time a person has had a Checking/Savings account, we would like to know the length of time you have had a Checking/Savings account.

As far as you can recall, approximately in which year did you open the Checking/Savings account that you currently have? If you currently have multiple accounts, consider the Checking/Savings account that you opened earliest.

() Decline to answer
Year of opening the account (4-digit, yyyy format): _____________

[Main questionnaire]

Now, we would like to understand your opinions regarding websites.

Imagine a scenario where you are a customer of a bank, and you have a Checking/Savings account with the bank. In this scenario, tell us how much you **agree or disagree** with the statements below. Use a scale from 0 to 10, with 0 indicating "strongly disagree" and 10 indicating "strongly agree."

Below, ***health-related browsing activity*** refers to browsing activities on websites such as WebMD, MedlinePlus or MedicineNet, which you might use/visit to find information on health conditions, symptoms or treatments.

[To minimize order effect, reverse the order of the 4 statements for half of the participants]

I want my bank to collect my health-related browsing activity to identify my financial needs and provide service relevant to me.

**strongly disagree 0**	**1**	**2**	**3**	**4**	**5**	**6**	**7**	**8**	**9**	**strongly agree 10**	Don't know/ Not sure	Decline to answer

I think my bank will collect my health-related browsing activity to identify my financial needs and provide service relevant to me.

**strongly disagree 0**	**1**	**2**	**3**	**4**	**5**	**6**	**7**	**8**	**9**	**strongly agree 10**	Don't know/ Not sure	Decline to answer

I deserve that my bank collect my health-related browsing activity to identify my financial needs and provide service relevant to me.

**strongly disagree 0**	**1**	**2**	**3**	**4**	**5**	**6**	**7**	**8**	**9**	**strongly agree 10**	Don't know/ Not sure	Decline to answer

I would tolerate if my bank must collect my health-related browsing activity to identify my financial needs and provide service relevant to me.

**strongly disagree 0**	**1**	**2**	**3**	**4**	**5**	**6**	**7**	**8**	**9**	**strongly agree 10**	Don't know/ Not sure	Decline to answer

[Post questionnaire]

[We get age range, household income, gender and US location for each panelist from SurveyMonkey]

You are almost done. Please tell us briefly about your background.

What is the highest level of school you have completed or the highest degree you have received?
() Less than high school (Grades 1-8 or no formal schooling)
() High school incomplete (Grades 9-11 or Grade 12 with NO diploma)
() High school graduate (Grade 12 with diploma or GED certificate)
() Some college, no degree (includes some community college)
() Two-year associate degree from a college or university
() Four-year college or university degree/Bachelor's degree (e.g., BS, BA, AB)
() Some postgraduate or professional schooling, no postgraduate degree (e.g. some graduate school)
() Postgraduate or professional degree, including master's, doctorate, medical or law degree (e.g., MA, MS, PhD, MD, JD, graduate school)
() Decline to answer

Please give us your feedback (optional).

If you had difficulty answering any of the survey questions, briefly describe what about the question made it difficult to answer (optional).

We are done with our questions. Anything you care to add? (optional).

Thank you very much for helping us with our study.
